# Knee Osteoarthritis: Current Insights Into Pathophysiology and Non-surgical Management Options

**DOI:** 10.7759/cureus.95302

**Published:** 2025-10-24

**Authors:** Mohammed Elmajee, Mahmoud Mersal, Bibi Zehra, Walid Ben Nafa, Ahmed Elsayed, Osama Embaby, Ahmed Elbioumy, Ahmed Elmahdi, Abdelrahman Embabi, Mohamed Youssef

**Affiliations:** 1 Spines, Royal Orthopaedic Hospital NHS Foundation Trust, Birmingham, GBR; 2 Trauma and Orthopaedics, University Hospitals Birmingham NHS Foundation Trust, Birmingham, GBR; 3 Surgery, Good Hope Hospital, University Hospitals Birmingham NHS Foundation Trust, Birmingham, GBR; 4 Trauma and Orthopaedics, St Helens Hospital, Merseyside, GBR; 5 Trauma and Orthopaedics, Manchester Royal Infirmary, Manchester, GBR; 6 Orthopaedics, Mansoura University Hospital, Birmingham, GBR; 7 Genera Surgery, Countess of Chester Hospital NHS Foundation Trust, Chester, GBR; 8 Trauma and Orthopaedics, Salford Royal NHS Foundation Trust, Manchester, GBR; 9 Emergency Clinical Fellow, Royal Surrey NHS Foundation Trust, Guildford, GBR; 10 Orthopaedics, Stoke Hospital, Stoke on Trent, GBR

**Keywords:** cartilage degeneration, conservative management of knee deformity, disease-modifying osteoarthritis drugs, intra-articular injections, knee osteoarthritis, mesenchymal stem cells, platelet-rich plasma, synovial inflammation

## Abstract

Knee osteoarthritis (OA) is a highly prevalent degenerative joint disorder, characterised by the progressive breakdown of articular cartilage (AC), remodelling of subchondral bone, synovial inflammation, and osteophyte formation. These pathological changes collectively lead to chronic pain, joint stiffness, and functional impairment, significantly diminishing patients' quality of life.

This review synthesises contemporary evidence on the pathophysiological mechanisms underpinning knee OA and critically appraises the efficacy of current conservative treatment strategies. Non-pharmacological interventions, such as weight reduction and structured exercise programmes, have consistently demonstrated substantial benefits in alleviating pain and improving functional outcomes. Pharmacological therapies, notably non-steroidal anti-inflammatory drugs (NSAIDs), offer effective symptom relief, while intra-articular interventions, including corticosteroid and hyaluronic acid injections, provide short-term pain control; however, their long-term efficacy remains uncertain. Emerging treatments, such as disease-modifying osteoarthritis drugs (DMOADs), genicular artery embolisation (GAE), and anabolic agents, have shown promising preliminary results, although their precise roles within conservative management require further validation through robust clinical trials.

Despite the growing array of therapeutic options, significant challenges persist in optimising conservative management strategies for knee OA. The need for individualised, multimodal approaches that account for patient-specific factors is increasingly recognised.

This review highlights the importance of tailored management pathways and emphasises the urgent need for continued research to refine and advance non-surgical interventions for individuals living with knee OA.

## Introduction and background

Prevalence of knee osteoarthritis

**Figure 1:**
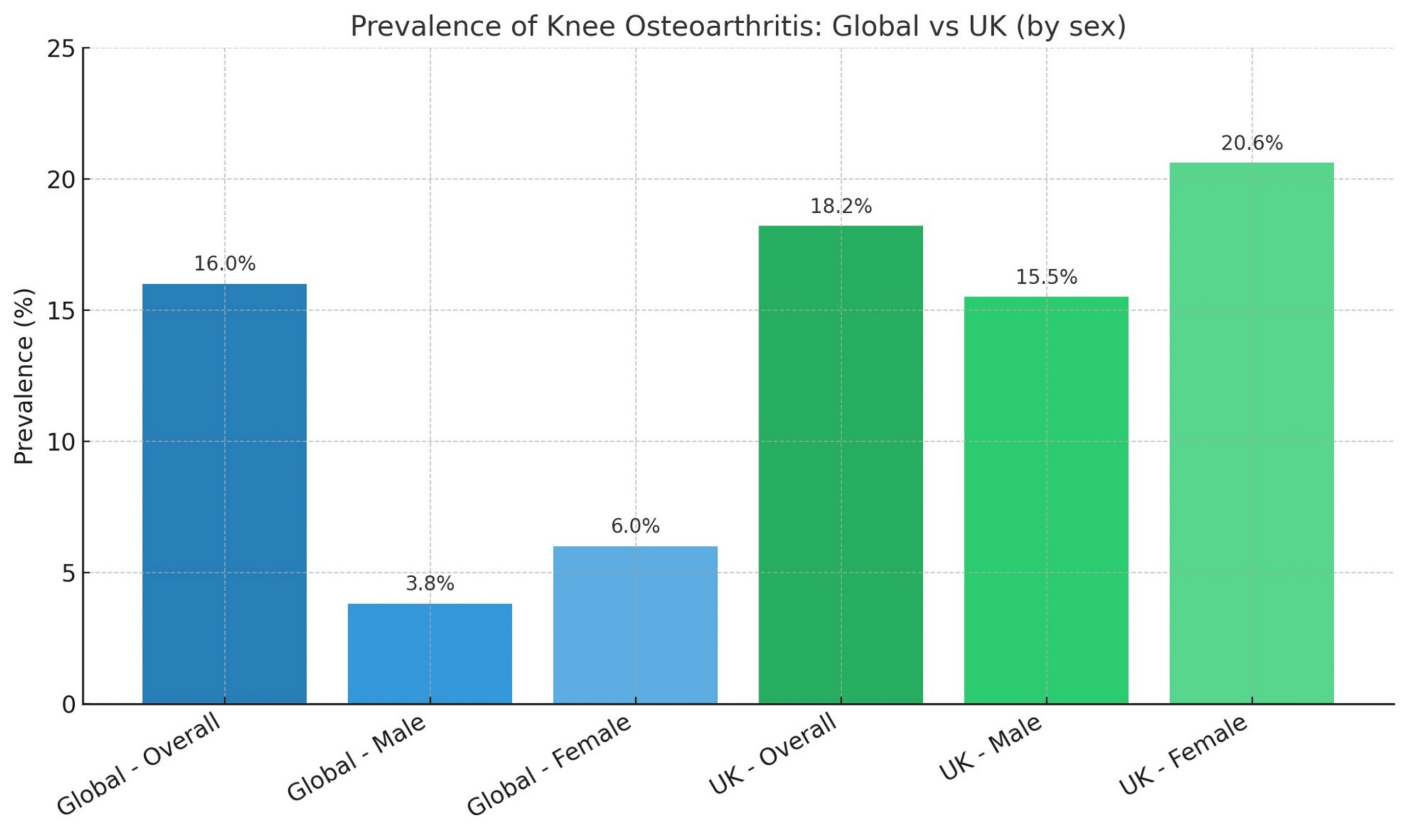
Global and UK prevalence of knee OA Chart created by the authors using data from [1,2]. OA: Osteoarthritis

Knee osteoarthritis (OA) is one of the most common musculoskeletal conditions, particularly affecting older adults. Globally, knee OA affects over 250 million people, making it a leading cause of disability, as illustrated in Figure 1 [1]. Its prevalence increases with age, with up to 10% of men and 13% of women over the age of 60 suffering from symptomatic knee OA [1]. The disease is multifactorial, with risk factors such as ageing, obesity, female gender, previous joint injury, genetic predisposition, and joint overuse contributing to its development [2].

The prevalence of knee OA is influenced by several risk factors, including: age (the risk of developing knee OA increases substantially after the age of 50); gender (women, particularly postmenopausal women, have a higher incidence of knee OA); obesity (being overweight or obese is one of the strongest modifiable risk factors, as excess weight places additional mechanical stress on the knee joints); and previous injury (people who have sustained knee injuries (e.g. ligament tears, fractures, etc.) have an increased risk of developing knee OA).

Future Projections for Knee OA in the United Kingdom and Across the Globe

The prevalence of knee OA is projected to rise significantly in the United Kingdom (UK) over the coming decades, driven by ageing demographics and increasing obesity rates. Understanding the future burden of knee OA in the UK is essential for planning healthcare resources and developing preventive strategies.

Ageing Population and Increased Incidence of Knee OA

As with many developed nations, the UK is experiencing a demographic shift towards an older population. The Office for National Statistics (ONS) predicts that by 2050, nearly a quarter of the UK population will be aged 65 and over [3,4]. Since knee OA is primarily age-related, this ageing trend is expected to lead to a significant rise in the number of individuals suffering from the condition. As per the recent prevalence estimates: currently, around 18% of people aged 45 and over in the UK have knee OA. By 2040, the prevalence is expected to increase by at least 50%, meaning millions more individuals will be affected [3,4]. In addition, in symptomatic knee OA, studies suggest that the proportion of older adults with symptomatic knee OA will also increase. Projections estimate that by 2035, over 8 million people in the UK could be living with symptomatic knee OA, nearly double the current number [4].

Impact of Obesity on Future Knee OA Prevalence

Obesity is a well-known risk factor for knee OA due to the increased mechanical stress on weight-bearing joints like the knees. The obesity epidemic in the UK is projected to significantly worsen the burden of knee OA. Approximately 28% of adults in the UK are classified as obese, with 36% overweight. This proportion is expected to rise in the coming years, especially among younger age groups [4]. Similarly, the increasing rates of obesity worldwide also play a key role in the rising incidence of knee OA across the globe. By 2030, nearly 86 million adults in the United States (US) are expected to be obese, a significant risk factor that will likely increase the prevalence of knee OA by 50% [5]. Furthermore, by 2050, it is estimated that 60% of men and 50% of women in the UK could be obese [4]. This would dramatically increase the prevalence of knee OA, as excess body weight is a major contributor to joint degeneration. A study suggested that for every 5 kg increase in body weight, the risk of knee OA rises by 36% [6].

Increased Demand for Knee Replacement Surgery

**Figure 2:**
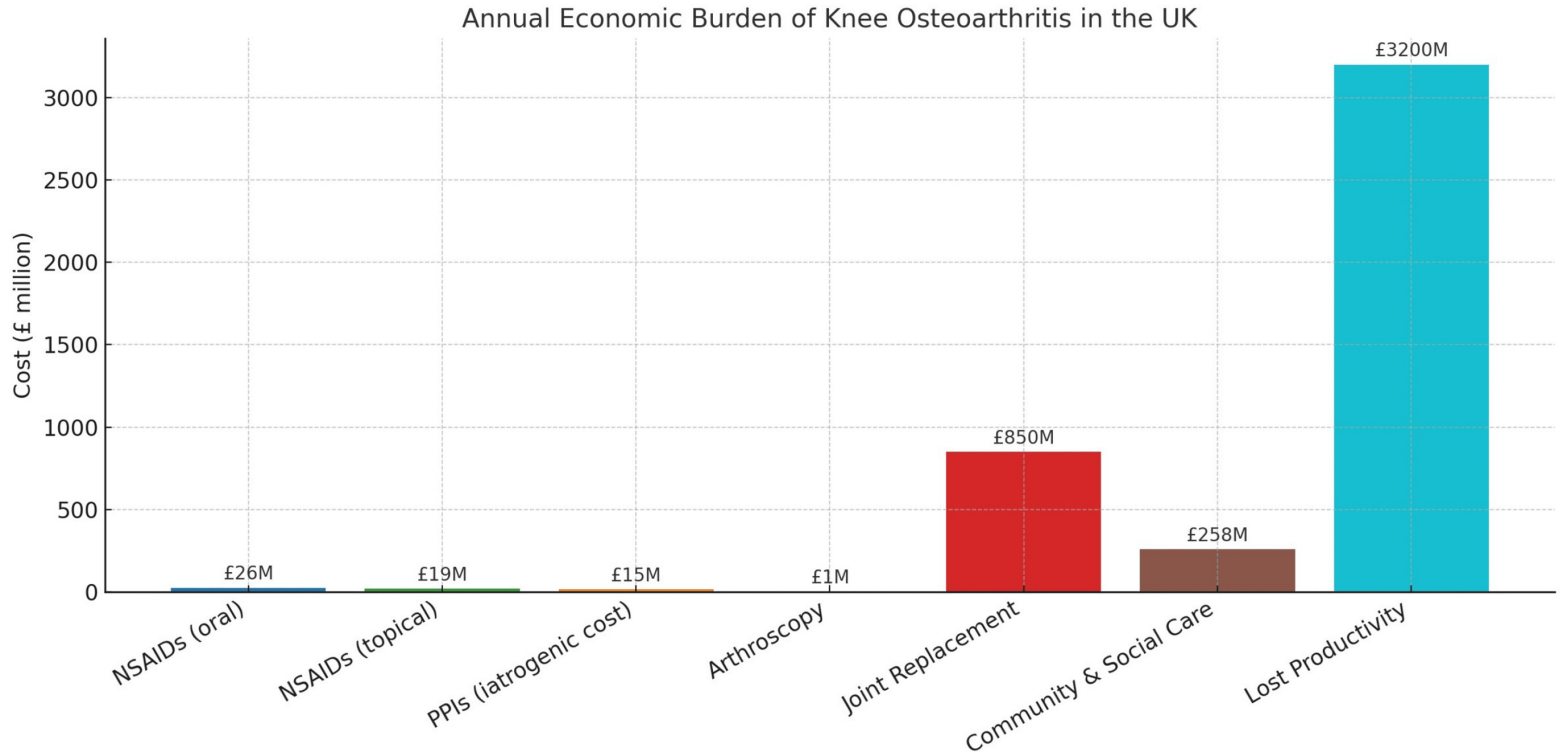
Annual economic burden of knee OA in the UK Figure created by the authors using data from [6,7]. OA: Osteoarthritis

With the anticipated rise in knee OA prevalence, the demand for surgical interventions, particularly total knee replacement, will also increase. Total knee replacement is often the final option for individuals with severe OA who have not responded to conservative treatments. Currently, over 100,000 knee replacement surgeries are performed annually in the UK, and that costs more money in compare to non-surgical treatment, and this is expected to increase dramatically [4]. Moreover, by 2030, the number of knee replacements is expected to rise by at least 60% due to the ageing population and growing obesity rates. This would result in up to 160,000 knee replacement surgeries per year [4]. The increased demand for total knee replacement surgeries will place a significant financial burden on the National Health Service (NHS). By 2035, the annual cost of knee OA management, including surgeries, is projected to exceed £3.5 billion, highlighting the need for effective preventive strategies and resource planning [4]. On a similar note, the number of total knee replacements is expected to increase by 673% between 2005 and 2030 in the US alone, reflecting the anticipated rise in disease burden (Figure 2) [5]. Data represent estimated annual costs of pharmacological management, surgical interventions, and indirect expenses in the UK. 

Health and economic burden

The growing prevalence of knee OA in the UK will lead to a substantial increase in the overall health and economic burden associated with the condition. These expenses are related to different aspects of knee OA treatment such as the cost of medications, physical therapy, and surgeries. The financial burden on the NHS is expected to grow, as knee OA management is already a significant contributor to healthcare expenditure [4]. By 2030, knee OA-related healthcare costs are predicted to exceed $250 billion globally, emphasising the importance of finding effective preventive measures [7]. Furthermore, OA is a leading cause of disability and work loss. As more individuals in the working-age population develop OA, the indirect costs associated with lost productivity and absenteeism is expected to increase significantly [4].

Challenges in Managing the Future Burden of Knee OA

The projected rise in knee OA cases will present significant challenges for both the UK healthcare system and the international community. Increasing patient numbers are expected to place further pressure on healthcare services, resulting in delayed diagnoses and restricted access to timely treatment. Within the NHS, this trend is already reflected in growing waiting lists for elective surgeries, including knee replacements. The expanding burden of OA will also strain available resources and workforce capacity, particularly among specialists, surgeons, and rehabilitation services. Moreover, widening socioeconomic disparities may exacerbate health inequalities, as individuals from lower-income backgrounds, who often have higher rates of obesity, face greater barriers to accessing effective care [2]. Pharmacological treatments remain limited in efficacy, offering only modest pain relief and carrying risks of gastrointestinal (GI), cardiovascular, and renal side effects with long-term use [3]. Despite extensive research, no FDA-approved disease-modifying therapies currently exist to halt or reverse OA progression, though several disease-modifying osteoarthritis drugs (DMOADs) are under investigation [3]. While total knee replacement remains an effective intervention for advanced disease, it is invasive, costly, and not suitable for all patients due to comorbidities or surgical risk. Furthermore, revision surgeries may be necessary in cases of implant failure, adding further complexity and cost to long-term management [1].

Pathophysiology of Knee OA

**Figure 3:**
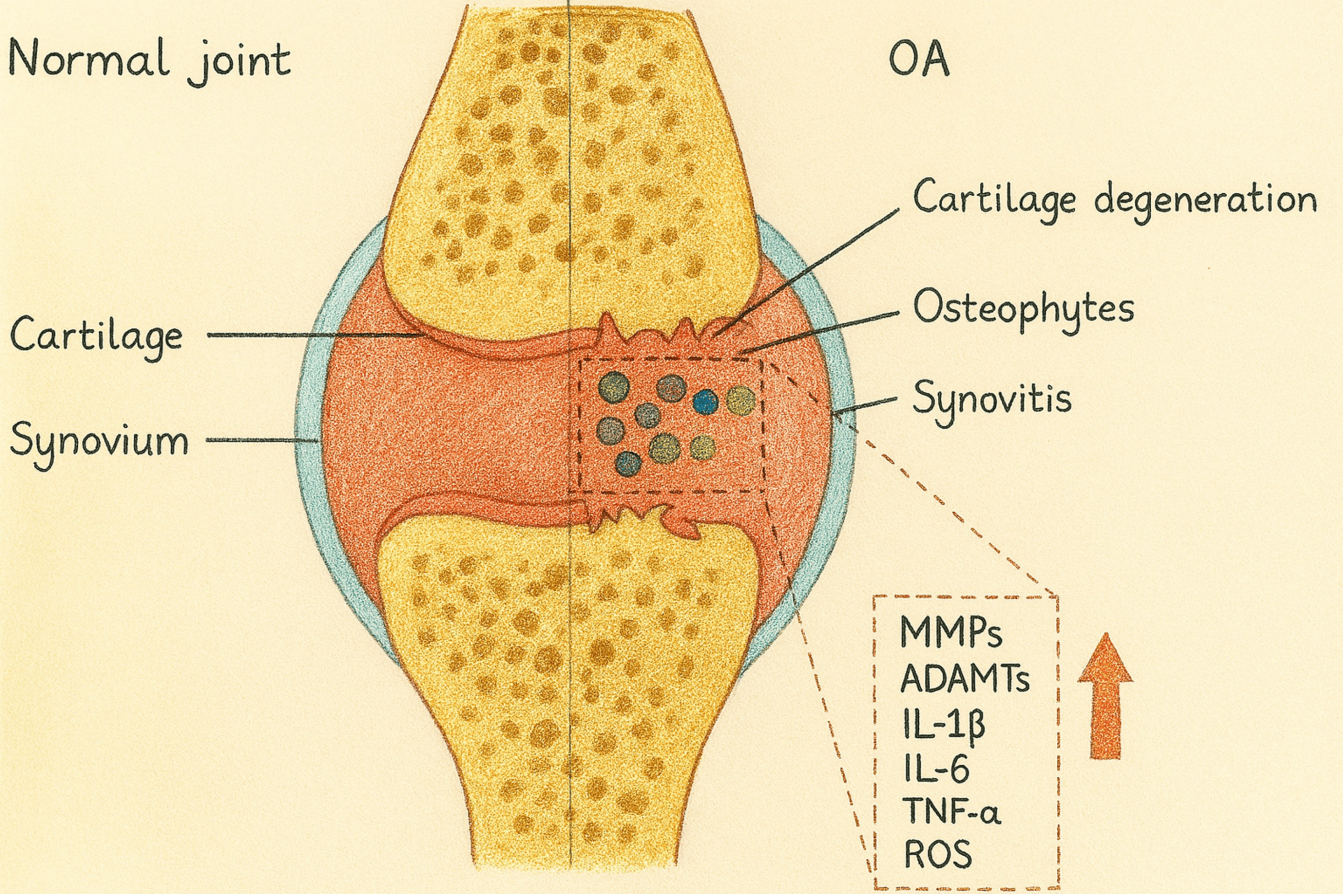
Comparison between a normal and osteoarthritic knee joint Figure created by the authors using original hand-drawn and edited in Adobe Illustrator (Adobe Inc.).

OA is a complex disease characterised by the degradation of articular cartilage (AC), subchondral bone remodelling, and synovial inflammation (Figure 3). No single definition encapsulates all manifestations of OA, as it results from a variety of pathways influenced by risk factors such as age, obesity, genetics, and mechanical stress [8]. The disease progresses along a continuum from early molecular changes to severe structural damage. While structural abnormalities are a hallmark, symptoms such as pain and stiffness often poorly correlate with imaging findings, reflecting the influence of systemic comorbidities, pain-processing mechanisms, and psychological factors on disease expression [9]. Over the recent years, OA has been viewed as a whole-person and whole-joint disease with all the tissues in or around the joint are influenced by the disease [8,9]. 

Knee Joint Changes

The AC is an avascular, alymphatic, and aneural tissue composed of extracellular materials (ECM) and cellular components. The cellular components, chondrocytes, the only cell type within its structure, which plays a pivotal role in maintaining cartilage integrity [10,11]. The ECM of AC is composed of over 70% water and a variety of organic components, including type II collagen, aggrecan, glycosaminoglycan, glycoproteins, and other proteoglycans. Additionally, it contains various types of collagens, including types III, VI, IX, and XI, which contribute to its structural stability [5,10,12]. Proteoglycan aggregates, which consist of negatively charged glycosaminoglycan such as keratin sulfate and chondroitin sulfate linked to aggrecan core proteins and hyaluronic acid, are enmeshed in a network of cross-linked type II collagen fibrils, ensuring cartilage resilience and mechanical support [11]. 

Defects in the aggrecan gene, such as premature termination codons, adversely affect cartilage development and may have secondary impacts on type II collagen, highlighting a feedback mechanism between ECM and collagen regulation [13-15]. While type II collagen and aggrecan are the predominant proteins in the ECM, the peri-cellular matrix surrounding chondrocytes contains unique other proteins like collagen VI, fibromodulin, and matrilin-3, which are essential for the localised microenvironment of these cells [16].

Chondrocytes maintain the delicate balance between the ECM synthesis and degradation through an intricate interplay of anabolic and catabolic processes. Mechanical loading plays a crucial role in regulating this equilibrium, as mechanoreceptors on the chondrocyte surface, such as mechanosensitive ion channels and integrins, sense mechanical stimuli and initiate intracellular signalling pathways. These pathways drive the biosynthesis of the ECM molecules, ensuring tissue integrity and repair [17,18]. Moderate mechanical loading, particularly through dynamic compression, can suppress the activity of proteolytic enzymes, maintain metabolic balance, and prevent cartilage damage. However, insufficient mechanical stimuli, such as immobilisation, can reduce the AC thickness by over 10% and lead to cartilage softening. On the other hand, excessive loading can disrupt the anabolic-catabolic balance, deplete matrix components, and result in irreversible cartilage destruction due to the limited regenerative capacity of the AC [17,19,20].

Knee OA is a whole-joint disease and all tissues inside and around the knee are affected during one stage of the disease process, including the subchondral bone [17]. Subchondral bone consists of two distinct layers: the cortical layer situated beneath the calcified cartilage, often termed the subchondral bone plate; and a deeper trabecular or cancellous layer [21]. The structure of subchondral bone is regulated by two primary cell types, osteoblasts and osteoclasts, in response to the local mechanical and biochemical environment, sharing similarities with chondrocytes, resulting in a significant role in subchondral bone remodelling [22]. Both subchondral bone plate and trabeculae undergo different biomechanical changes at different stages of OA as a result of the changes to the equilibrium between different cell types, particularly osteophytes and osteoblasts, resulting in bone turnover changes such as subchondral bone lesions, cysts and osteophyte formation [17]. 

Synovial tissue and fluid changes is another manifestation of osteoarthritic process in knee OA. The synovial membrane consists of two layers: the intima, a thin lining layer; and the sub-intima, a vascularised connective tissue layer. In healthy individuals, the synovial lining should be less than 5 mm thick. The intima contains synoviocytes, which include macrophage-like and fibroblast-like cells. Macrophage-like synoviocytes contribute to immune responses, while fibroblast-like synoviocytes secrete hyaluronic acid and other matrix components [23,24]. These cells maintain synovial fluid volume and produce key molecules like lubricin and hyaluronic acid to ensure joint lubrication and functionality [24]. Synovial hypertrophy and thickening in synovitis is associated with inflammatory mediator activity, particularly interleukins (IL-1, IL-6, and IL-17) and tumour necrosis factor (TNF) [25]. These factors stimulate synoviocytes and chondrocytes to produce degradative enzymes and prostaglandins, perpetuating inflammation and cartilage destruction. These changes, combined with heightened neuronal sensitivity, underscore the progressive nature of pain and dysfunction in the knee OA [25].

Other tissues are affected by the OA changes in the knee, the infrapatellar (Hoffa’s) fat pad (IFP) is an adipose tissue structure in the anterior knee compartment, located between the synovial and capsular layers, and richly vascularised and innervated [26]. The IFP contains lots of different cells, majority of them without fully understood role, produces and releases cytokines, ILs, and growth factors by means of paracrine, endocrine, and autocrine mechanisms [27]. In the knee OA, the IFP undergoes histological and biochemical changes such as increased number of macrophages and pain-inducing substances, for example, substance P [28]. These changes will not lead to structural changes within the the IFP itself only but also lead to increase in the production of adipokines and cytokines such as adiponectin, leptin, IL-6 and TNF [29]. For instance, high intra-articular leptin levels have been correlated with the increased severity of OA changes [29]. 

OA changes not only affect different tissues in and around the knee but also there are a number of different chemical and biochemical factors which have impact on the disease progress and changes. The immune response and the release of different mediators such as cytokines play a critical role in the pathogenesis of knee OA [30]. Cytokines are categorised as inflammatory (e.g., IL-1β, TNF-α, IL-6) or anti-inflammatory (e.g., IL-10, IL-13), with inflammatory cytokines promoting catabolism and cartilage breakdown [30,31]. The inflammatory cytokines activate different pathways which ultimately induce enzymes such as matrix metalloproteinase (MMPs) that degrade ECM [31]. Anti-inflammatory cytokines like IL-10 modulate these effects, reducing cartilage loss. Cytokine profiles differ between joints and OA subtypes, correlating with OA severity and symptoms associated with OA highlighting their diagnostic and therapeutic potential and biomarkers for early OA detection and progression monitoring, opening avenues for personalised treatments targeting these inflammatory pathways [32-35]. 

The prevalence of obesity has reached pandemic proportions globally [36]. Obese individuals (BMI > 30; excessive body fat accumulation that presents a risk to health) are four times more likely to experience knee OA compared to controls [37]. Furthermore, perioperative complications such as superficial wound infection and periprosthetic joint infection have been demonstrated to be higher in obese individuals [38]. In addition, patients' revision rate of total knee arthroplasty in morbidly obese patients (BMI higher than 40) is increased relative to non-obese (7% vs. 2%) [38]. Mechanical factors and stress associated with obesity have always been linked to OA development and progression, but metabolic factors have been postulated as an associated means to explain OA pathogenesis in obese individuals [39,40]. Adiponectin, resistin, leptin and visfatin are adipokines associated with OA, either released from local adipose tissues, such as the IFP, or from the systemic circulation [41,42]. 

The pathogenesis of OA in the knee and other joints is complex, and it is difficult to explain by one or two factors with interactions between different factors, mechanical, chemical, metabolic and genetic factors have been described. Around 90 genetic loci have been detected as loci for OA development [39]. MicroRNA (miRNAs) are a family of non-coding RNAs (ncRNAs) that bind to mRNA and regulate gene expression by modifying protein synthesis [39]. miRNAs are ncRNAs that exhibit an epigenetic mechanism which in turns regulates gene expression. miRNAs are also regulated by common epigenetic mechanisms, including DNA methylation, histone modification, and RNA modification, forming a complex feedback loop that significantly impacts gene expression [40]. A research suggests that disruptions in this regulatory loop may play a crucial role in the development of various diseases, including OA [40].

## Review

Management for knee OA

Overview

Over the last five decades, overall treatment strategy for knee OA has not drastically changed, with knee replacement surgery being the definitive and final option available when conservative treatment fails. Given the increasing number of OA cases worldwide and the relative ineffectiveness of conservative management in comparison to surgical intervention, there is a pressing need for further research to explore innovative treatment strategies, improve early diagnosis, and develop more effective non-surgical therapies to better address this growing global health challenge. 

Knee OA is associated with several symptoms, with pain being the most prominent. Pain is a multifaceted phenomenon that likely arises from the interaction of various mechanisms and factors, encompassing both peripheral (knee-specific) and central components [43-45]. Peripherally, different tissues within and surrounding the knee contribute to pain generation, with nociceptor stimulation driven by factors such as cytokines, adipokines, and other inflammatory mediators being the primary pain generators [45,46]. Furthermore, these mediators drive synovial angiogenesis, and proangiogenic factors are known to stimulate nerve growth [47]. Centrally, mechanisms involving the spinal cord and brain play a significant role in modulating the pain experience and threshold in patients with knee OA. These central mechanisms include increased excitability and reduced inhibitory processes at the spinal or cortical levels, a phenomenon often described as secondary hyperalgesia, which involves heightened sensitivity of neurons in the dorsal horn of the spinal cord [45].

Conservative management strategies are the first line of treatment, aiming to alleviate symptoms and improve quality of life. These interventions encompass different strategies including physical therapy, pharmacological treatments (analgesics, anti-inflammatory and others), weight management, injection therapies (intra-articular and genicular artery embolisation (GAE) therapies) and lifestyle modifications. 

Physical therapy

Various types of physical therapies have been described for managing knee OA, each exhibiting varying levels of effectiveness. Current guidelines and contemporary literature emphasise the combination of strengthening, aerobic, and functional exercises as a recommended approach [48,49]. These exercise therapies have demonstrated the ability to reduce pain and improve function and overall health status in individuals with knee OA [48]. The prescription of both general exercises, such as aerobic fitness training, and localised exercises, such as muscle strengthening, is strongly encouraged. Crucially, exercise therapy should be individualised and patient-centred, with careful consideration of factors such as age, comorbidities, and overall mobility [49]. To optimise outcomes, key parameters, including the type of exercise (aerobic, performance-based, or resistance); frequency; mode; dosage; setting (e.g., home-based or clinic-based) and delivery method (self-administered or supervised), should be tailored to the patient’s specific needs and circumstances [50]. Adherence to prescribed exercise regimens remains the principal predictor of long-term success, highlighting the importance of strategies to promote sustained engagement [50]. By customising therapy to the individual and fostering adherence, exercise interventions can maximise their effectiveness in managing knee OA symptoms. Various mechanisms have been identified through which different types of exercise can induce molecular, physiological, and genetic changes that may interrupt the progression of knee OA [51]. The majority of these mechanisms have been demonstrated using either animal models or in vitro studies. They include inhibiting ECM matrix degradation, preventing apoptosis and preserving AC, mitigating inflammatory responses by modulating signalling pathways to potentially delay OA progression, and regulating the expression of ncRNAs, which in turn influence the AC, synovial membrane, and subchondral bone in OA patients [52-54].

One of the important concepts that is required to be discussed with patients with knee OA is the “vicious-cycle pain concept”. Timing and intensity of physical therapies prescribed or advised should be carefully considered [55]. Although exercising while in pain may appear counterintuitive, an increasing body of evidence underscores the significance of initiating low-impact activities and gradually progressing the intensity and type of physical exercises [55,56]. This approach involves a steady and consistent increase in physical activity levels while carefully acknowledging and managing the degree of pain experienced.

The effectiveness of Kinesio Taping (KT) in managing knee OA has been a subject of debate over the past two decades since its introduction in the early 2000s [57,58]. Despite this controversy, KT has been shown to alleviate pain and improve physical function in individuals with knee OA [57]. Evidence also suggests that KT is superior to other interventions in enhancing muscular strength. Additionally, potential benefits such as its psychological impact and the improvement of knee joint stability have been proposed [59]. However, the application of KT requires careful consideration to ensure its appropriateness and effectiveness, particularly with regard to the duration and severity of the disease. Prospective, well-controlled trials are necessary to further evaluate the efficacy of KT in the management of knee OA, addressing existing gaps in evidence and providing a more definitive understanding of its role in clinical practice.

Knee orthosis and insoles

Knee braces, another means to manage, are widely classified into various categories, offering a range of designs that complicate the selection of an appropriate treatment for patients with knee OA [50]. The effectiveness of knee braces depends on several factors, including both patient-specific and brace-specific characteristics [60]. For instance, braces have been shown to be less effective in obese patients, while custom-made braces outperform standard "off-the-shelf" designs in terms of efficacy and comfort. The frequency and duration of brace use vary significantly among patients, which impacts treatment outcomes. Adherence to brace use remains one of the primary challenges in maximising their therapeutic benefits. Studies have reported that 42-50% of patients discontinue brace use within six months, highlighting the importance of designing braces that encourage consistent wear [61]. Custom-fitted unloader knee braces with valgus and external rotation functions have demonstrated superior outcomes compared to traditional valgus three-point bending systems [62]. These braces provide better comfort, more frequent daily use, and greater reductions in knee adduction moments, both before and after treatment [62]. For optimal effectiveness, knee braces must not only deliver functional benefits but also ensure a proper fit, high comfort, a slim profile, and ease of adjustment to promote patient adherence to prescribed treatments [63]. The use of insoles and specialised footwear for treatment of knee OA, particularly isolated compartment knee OA has been explored in the literature, yielding mixed results. While some studies report positive and encouraging outcomes, others present less favourable findings [64,65]. Similar to knee braces, the effectiveness of insoles is influenced by a variety of factors, including their characteristics, such as type, height, length, inclination angle, and material composition, as well as patient-specific variables, such as the grade and stage of OA, compliance, and BMI [50]. To conclusively determine the efficacy of different insoles and footwear, long-term, robust, randomised, and well-controlled clinical trials are needed.

Pharmacological management

Different pharmacological agents have been utilised over more than half a century to manage symptoms related to knee OA. Clinicians should take multiple factors into account to optimise personalised treatment plans, including risk factors, potential toxicities, comorbid conditions, and interactions with other medications. Each medication should undergo a thorough risk-benefit analysis, which should be discussed with patients to encourage informed and collaborative decision-making. Additionally, patient outcomes should be regularly monitored.

Topical Therapies

Topical treatment options can be divided into pharmacological and physical topical options. Three topical pharmacologic therapies are mentioned in some clinical guidelines; capsaicin, topical NSIADs and salicylates. Capsaicin is occasionally used as a topical analgesic for knee OA. The American College of Rheumatology (ACR) conditionally recommends its use for knee OA [66]. Capsaicin is applied topically for OA, where it is absorbed through the skin but does not significantly enter systemic circulation due to its lipophilic nature [67]. Following the use of a high-dose patch, its elimination half-life averages 1.64 hours, although this varies with concentration and formulation [67]. For instance, a 3% topical solution has a longer half-life of approximately 24 hours suggesting slow metabolism and biotransformation in human skin [67]. Capsaicin, a derivative of chili peppers, is a transient receptor potential vanilloid (TRPV) agonist [68]. TRPV receptors respond to heat, acidity, and abrasion [68]. Capsaicin reduces TRPV’s activation threshold and induces a prolonged refractory state, leading to “de-functionalisation” of nociceptor fibres, which reduces pain [68]. It also blocks the neuronal transport of neuropeptides such as substance P and somatostatin, thereby lowering their availability and contributing to its analgesic effects [68]. Topical capsaicin is associated with mild, localised adverse effects, including transient pain, redness, itching, and swelling [67]. Systemic effects, such as hypertension, nausea, and vomiting, are rare. Clinical trials report small, temporary increases in arterial pressure, potentially linked to pain during application [67].

Topical non-steroidal anti-inflammatory drugs (NSAIDs) are sometimes favoured over oral formulations for knee OA due to lower systemic exposure and reduced toxicity risk [69]. Some guidelines are strongly recommending their application for knee OA and conditionally for polyarticular OA without comorbidities, as well as for patients with GI, cardiovascular, or frailty-related conditions, owing to their modest pain relief and minimal localised side effects, such as skin irritation [66]. For knee OA with widespread pain or depression, different guidelines conditionally recommend the use of topical NSAIDs but not advised for polyarticular OA in similar cases [66,69]. Some guidelines caution against exceeding the total NSAID dose when combining topical and oral forms. Unlike oral NSAIDs, topical formulations achieve therapeutic concentrations in local tissues (e.g., synovium and cartilage) while maintaining plasma levels below 10% of oral administration, thereby minimising systemic effects [70]. Common side effects include dry skin, redness, and itching, with occasional mild systemic issues like GI discomfort or headaches [71]. 

Salicylates, while chemically related to NSAIDs and sharing a mechanism of cyclooxygenase (COX) inhibition, are recognised as a distinct therapeutic class with unique clinical effects [72]. Their pain-relieving action is primarily attributed to counter-irritation [72]). This process involves stimulating sensory nerve endings, leading to localised irritation that helps to modulate or diminish pain signals originating from underlying joints served by the same nerve pathways. By creating a competing stimulus, salicylates may disrupt the perception of pain, providing symptomatic relief in musculoskeletal conditions [72]. Additionally, their skin-irritating properties contribute to this counter-irritant effect, further distinguishing them from traditional NSAIDs [73].

Physical topical therapies encompass a variety of superficial heating or cooling treatments, as well as contrast therapy, each targeting pain relief and recovery. These methods are typically delivered through practical and accessible tools, such as circulating water systems, electric heating pads, warm towels, ice packs, or ice massages [73]. Cooling, or cryotherapy, is frequently employed in rehabilitation settings to mitigate inflammation, pain, and swelling following injuries or in chronic conditions such as knee OA. The application of cold constricts blood vessels limiting inflammation and associated swelling. Additionally, cooling numbs the affected region by blocking nerve impulses, offering temporary pain relief [73]. This method is particularly effective in the acute phase of injuries, such as sprains or strains, and is a cornerstone of the RICE (Rest, Ice, Compression, and Elevation) protocol.

Heat therapy, on the other hand, is beneficial for promoting relaxation, reducing muscle tension, and increasing circulation to the targeted area. The application of warmth dilates blood vessels, enhancing the delivery of oxygen and nutrients while facilitating the removal of metabolic waste. This improved circulation supports tissue repair and relieves stiffness, making it an effective treatment for chronic conditions such as knee OA or muscle spasms. Heat therapy is often combined with other treatments, such as physical therapy or medication, to optimise outcomes. Contrast therapy alternates between hot and cold applications, typically in timed intervals, to potentially combine the benefits of both therapies [73]. The rapid changes in temperature are thought to stimulate blood flow and lymphatic drainage, enhancing circulation and reducing swelling. However, the exact mechanisms and efficacy of contrast therapy remain unclear, with limited robust evidence to confirm its superiority over isolated heat or cold therapies [74]. Despite this, it continues to be used in rehabilitation for its purported benefits in promoting recovery and reducing pain. These physical topical therapies provide non-invasive and generally safe options for managing pain and inflammation, though their effectiveness may vary based on the condition being treated and individual patient response. Further research is needed, particularly on the mechanisms and outcomes of contrast therapy, to better inform clinical practice.

Oral Medications

There are different pharmacological agents that can be utilised to alleviate pain, reduce inflammation, and slow disease progression of knee OA. These tablets are usually part of multifaceted management approaches commonly applied to treat knee OA. These agents include acetaminophen, tramadol, NSAIDs, steroids, duloxetine, supplements like glucosamine and chondroitin sulfate and DMOADs. 

Acetaminophen is widely used for knee OA pain management, although its efficacy remains a subject of debate, and guidelines vary in their recommendations for its use in knee OA [75]. Despite more than 60 years of widespread use, acetaminophen's precise mechanism of action is not definitively established. However, it has been shown to activate serotonergic pathways and weakly inhibit prostaglandin synthesis, thereby affecting central and peripheral pain processes, although it lacks the anti-inflammatory properties of NSAIDs [76]. Acetaminophen is rapidly absorbed, with a systemic availability of 70-90%, low plasma protein binding (10-25%), and a half-life of two-three hours [76]. It is primarily metabolised in the liver and excreted in urine, with 90% eliminated within 24 hours [76]. While generally safe at therapeutic doses, prolonged or high-dose use can lead to severe hepatic toxicity, renal tubular necrosis, hypoglycaemic coma, and thrombocytopenia [76].

Tramadol, a synthetic opioid and serotonin norepinephrine reuptake inhibitor, can be used to manage knee OA pain, particularly in patients with contraindications to other treatments or limited therapeutic options, as conditionally recommended by some clinical guidelines [75]. Its mechanism of action involves two active enantiomers: (+)-tramadol, which acts as a μ-opioid receptor agonist and inhibits serotonin reuptake, and (−)-tramadol, which inhibits norepinephrine reuptake [77]. Tramadol also modulates pain through interactions with NMDA, GABA (A), muscarinic receptors, and prostaglandin inhibition [77]. When administered orally, it has a bioavailability of 68%, reaching peak serum concentrations within two hours, with an elimination half-life of five hours for tramadol and eight-nine hours for its primary metabolite, M1 [77]. It is metabolised in the liver and primarily excreted via the kidneys, with 30% excreted unchanged in urine. Adverse effects, which are dose-dependent, include nausea, headache, dizziness, and drowsiness, while severe effects may include respiratory depression and serotonin syndrome [77]. Tramadol’s use in OA has raised concerns due to associations with increased risks of mortality, venous thromboembolism, and hip fractures compared to NSAIDs, though fatal intoxications are rare due to its weaker opioid potency [77,78].

Duloxetine, a serotonin and norepinephrine reuptake inhibitor is conditionally recommended by various guidelines for managing knee OA, particularly in patients with coexisting depression [79]. While not specifically addressed in guidelines, duloxetine is also widely used for OA-related neuropathic pain [80]. The medication modulates central nervous system pain through serotonergic and noradrenergic pathways in descending spinal pathways, although the exact mechanisms remain unclear [75]. Duloxetine is administered orally, is well absorbed, and reaches peak plasma concentrations within six hours [81]. It crosses the blood-brain barrier and accumulates in the cerebral cortex. Its elimination half-life is approximately 12 hours, with metabolism occurring in the liver and excretion primarily through urine (70%) and faeces (>20%) [81-83]. Concerns regarding toxicity include hepatotoxicity, which can result in liver injury with elevated transaminase and bilirubin levels [83]. Other risks include orthostatic hypotension, syncope, blood pressure increases, and rare reports of mania, hypomania, or seizures [84]. Common side effects include dry mouth, constipation, nausea, fatigue, decreased appetite, drowsiness, and dizziness [83].

Oral NSAIDs remain a widely utilised treatment option for knee OA-related pain, owing to their efficacy in managing both pain and inflammation [84]. However, their widespread use is tempered by concerns regarding toxicity, prompting careful consideration of patient risk profiles [79]. NSAIDs primarily exert their therapeutic effects through the inhibition of COX enzymes, specifically COX-1 and COX-2 [79]. These enzymes are responsible for the conversion of arachidonic acid into prostaglandins, which play a key role in the inflammatory response [79]. By inhibiting COX enzymes, NSAIDs effectively reduce the production of prostanoids, leading to anti-inflammatory, analgesic, and antipyretic effects [79]. Despite their effectiveness, clinicians must weigh these benefits against potential adverse effects. Non-selective NSAIDs inhibit both COX-1 and COX-2 enzymes, which, while reducing inflammation and pain, can lead to GI toxicity [79]. This includes gastric mucosal damage, nausea, ulceration, and severe complications such as GI haemorrhage [79]. Long-term use of non-selective NSAIDs is associated with an increased risk of nephrotoxicity, particularly in patients with underlying conditions such as heart failure, hypertension, and diabetes, where glomerular filtration rates may be compromised [75]. Additionally, the cardiovascular risks associated with NSAIDs are heightened during the initial month of use, with increased likelihood of acute cardiovascular events and heart failure [75,85]. For individuals with elevated cardiovascular or renal risks, it is recommended to use NSAIDs cautiously or avoid them altogether [75,85]. Pharmacokinetically, NSAIDs are rapidly absorbed from the GI tract, with high protein binding restricting their distribution to extracellular spaces [85]. They are extensively metabolised in the liver and excreted primarily through urine and bile. Given the balance between their therapeutic benefits and the potential for adverse events, careful monitoring and individualised treatment plans are crucial for the safe use of NSAIDs in managing OA [85].

Topical NSAIDs are increasingly preferred over oral formulations due to their potential for lower systemic exposure and reduced toxicity [66]. Various clinical guidelines emphasise the benefits of topical NSAIDs for managing knee OA-related pain, particularly due to their ability to target localised tissues with minimal systemic involvement [66]. For instance, the Osteoarthritis Research Society International (OARSI) strongly recommends topical NSAIDs for patients with knee OA [79]. Topical NSAIDs are particularly advantageous for patients with GI, cardiovascular, or frailty-related morbidities, as they provide modest pain relief with fewer adverse side effects, such as minor, transient skin reactions [66]. OARSI also highlights the importance of monitoring the number of joints treated and concurrent oral NSAID use to ensure that the recommended dosage is not exceeded [79]. Despite their effectiveness, topical NSAIDs produce minimal systemic absorption, reducing the risk of widespread adverse effects [66]. However, localised side effects such as dry skin, erythema, irritation, and occasional mild systemic effects like GI discomfort and headaches may still occur [79]. These benefits and limitations underscore the importance of tailoring topical NSAID use to individual patient needs while monitoring both localised and potential systemic side effects [66,79].

Selective COX-2 inhibitors are conditionally recommended by OARSI for managing knee OA in patients who do not present with comorbidities or in those with GI-related comorbidities [79]. However, these inhibitors are not recommended for individuals with cardiovascular comorbidities due to their association with heightened cardiovascular risks, including oedema, thrombosis, blood pressure destabilisation, and an increased risk of myocardial infarction [79]. OARSI also emphasises that combining selective COX-2 inhibitors with proton-pump inhibitors can enhance their safety profile by minimising GI risks, which are generally lower compared to non-selective NSAIDs [79]. Mechanistically, COX-2 inhibitors selectively block the COX-2 enzyme, which plays a pivotal role in the synthesis of prostaglandins, compounds responsible for inflammation and pain. By targeting COX-2, these inhibitors help reduce the production of inflammatory prostaglandins while preserving the cytoprotective effects of COX-1, which is essential for maintaining gastric mucosal integrity [86]. This selectivity results in decreased inflammation and pain with a reduced risk of GI complications such as ulcers and bleeding [66]. Pharmacokinetically, after oral administration, celecoxib, a commonly used COX-2 inhibitor, is rapidly absorbed and reaches peak serum concentration within approximately three hours [79]. It is extensively metabolised in the liver, with only a small percentage (around 2.6%) of the dose excreted through urine or faeces [86]. The liver processes celecoxib into inactive metabolites, ensuring minimal systemic exposure to active drug components [86]. Despite their reduced GI side effects compared to non-selective NSAIDs, selective COX-2 inhibitors carry increased cardiovascular risks, and renal complications may arise similar to those seen with non-selective NSAIDs [66]. These risks are influenced by alterations in sodium and potassium handling, renal blood flow, and water retention [79].

Injection therapies

Intra-articular Hyaluronic Acid

Intra-articular hyaluronic acid (IAHA) is commonly used to manage knee OA [87]. The OARSI conditionally recommends IAHA for all comorbidity groups, emphasising its potential benefits for pain relief and long-term safety compared to intra-articular corticosteroids (IACS) [79]. A recent meta-analysis demonstrated that patients experiencing early to mild knee OA, the available evidence indicates that IAHA demonstrates comparable effectiveness to other biologically active substances frequently administered into the knee joint [88]. This therapeutic similarity is observed for a duration of at least three months following the treatment [88]. Although the exact mechanisms of action of IAHA remain unclear, hyaluronic acid functions as both a structural and signalling molecule with disease-modifying effects [87]. High molecular weight hyaluronic acid injections may improve joint viscoelasticity, hydration, and structural integrity [87,89]. Additionally, hyaluronic acid can act as a signalling molecule influencing cell migration, cell division, inflammatory mediators, and tissue remodelling. It interacts with cell-surface receptors, such as CD44 and intracellular adhesion molecule 1, which affect collagen and matrix integrity [89]. IAHA has a short half-life ranging from 17 hours to 1.5 days, depending on its molecular weight, and is primarily metabolised through tissues, lymphatics, and vasculature [89]. IAHA demonstrates a relatively low toxicity profile, with minor, transient local reactions at the injection site being the most common adverse effects [87,88]. Based on the comprehensive review of available evidence, including recent high-quality research studies, some authors suggest that IAHA injections may offer benefits in managing early or mild knee OA, particularly in providing pain relief over a short-term period of several weeks to a few months. However, these injections are unlikely to yield significant long-term benefits or lead to substantial improvements in functional outcomes.

Intra-articular Corticosteroids

IACS are widely used for short-term pain relief in knee OA, typically providing symptom relief for less than four weeks [79]. The first IACS injection was conducted in knee OA in 1953 by Hollander [90]. Once injected, corticosteroids penetrate cell membranes and bind to nuclear steroid receptors, modulating gene transcription and translation [91]. This interaction suppresses immune responses and reduces inflammation by inhibiting pro-inflammatory mediators such as phospholipase A2 and IL-2 while decreasing arachidonic acid derivatives [91]. Additionally, corticosteroids downregulate COX-2 expression and prostaglandin synthesis while promoting the production of anti-inflammatory proteins like annexin A1 [91]. They also induce eosinophil and T-cell apoptosis, contributing to their immunomodulatory effects [92]. Emerging new evidence demonstrated that IACS injections have been shown to be significantly and clinically efficacious at reducing knee OA pain for at least one week [93]. Different formulations are available; however, triamcinolone appeared to be more efficacious than either betamethasone or methylprednisolone [93].

Modern corticosteroid formulations, including those incorporating nanomaterials or crystalline suspensions, enhance drug retention within joint tissues and prolong therapeutic effects [94]. Systemic effects typically last between one and four weeks, with inflammatory cytokines decreasing rapidly post-injection [91]. For instance, markers like C-reactive protein may show reductions lasting several days to months [95]. Corticosteroids are primarily metabolised in the liver, with plasma concentrations decreasing within 24-48 hours post-injection [95]. Elimination half-lives of conventional corticosteroids range from 8-72 hours, whereas extended-release formulations, such as microsphere triamcinolone acetonide, can achieve half-lives of up to 634 hours [96]. Despite numerous investigations that have been conducted to identify the optimal corticosteroid agent, and its optimal dosing regimen for the intra-articular treatment of knee OA, consensus regarding the optimal corticosteroid agent, dosing regimen, and injection technique remains elusive, as evidenced by variability in clinical practice across orthopaedic communities [95]. A survey conducted among members of the American Association of Hip and Knee Surgeons revealed a strong consensus regarding the use of IACS [97]. The findings indicated that while intra-articular corticosteroid injections (ICIs) are generally effective, their efficacy tends to diminish with repeated use [97]. Most respondents agreed on a minimum interval of three months between injections, although no definitive lifetime limit was established [97]. There was also significant agreement on maintaining a three-month gap before any surgical procedures [97]. However, variations were observed among American surgeons regarding the choice of steroid formulation, local anaesthetic agents, and skin preparation techniques [97]. Similarly, a recent survey exploring perspectives on ICIs among UK clinicians highlighted the perceived benefits of ICIs in alleviating symptoms and enhancing patients' quality of life [98]. The survey also underscored variability in dosing, frequency, and timing of injections, reflecting clinicians' uncertainty about existing guidelines [98]. Future, randomised, large studies are required to delve deep into those uncertainty, however, with regards to dosage there is some evidence that dosages equivalent to 40 mg of triamcinolone seem to be associated with a longer duration of pain relief (12-24 weeks) compared to lower dosages (two-four weeks) [99]. The choice of injection site also influences outcomes. A systematic review identified eight palpation-guided knee injection sites, with superomedial and superolateral patellar approaches achieving the highest accuracy rates (82% and 87%, respectively) [100]. Injector expertise further impacts accuracy, with less experienced clinicians achieving only 55% accuracy compared to 100% for experienced practitioners [100]. Ultrasound guidance may be required in cases of obesity, failed palpation-guided injections, or structural deformities affecting accuracy [101]. Despite its benefits, IACS use is associated with several adverse effects. Minor complications include injection site pain, local swelling, and skin changes [102]. Reports indicate that IACS injections may lead to small atrophic lesions at the injection site, subcutaneous tissue atrophy, and peri-articular or intra-articular calcifications following more than 18 months of follow-up [103]. Approximately 40% of patients experience flushing after IACS administration, with some cases involving serious reactions. Flushing typically occurs around 19 hours post injection, lasts for approximately 36 hours, is more prevalent in females, and tends to be more severe in patients receiving higher doses [104]. Additionally, complications such as transient hyperglycaemia and a cushingoid appearance may arise with excessively frequent IACS injections [105]. Up to 25% of patients may experience transient suppression of the hypothalamic-pituitary-adrenal (HPA) axis, evidenced by reduced blood cortisol levels post-injection, which generally return to baseline within one to four weeks [106]. Pseudoseptic reactions, characterised by joint inflammation and swelling without infection, occur in 1-3% of cases, particularly after repeated injections [102]. Cross-linked IACS preparations are associated with severe acute inflammatory reactions in 2-8% of patients compared to naturally-occurring IACS preparations [104,105]. This is likely due to the chemical cross-linking process utilised to prolong its half-life [105]. As these formulations differ in their mean molecular weight ranging from (500 to 6000 kDa), concentration (0.8-30 mg/mL), volume of injection (0.5-6.0 mL), source (animal versus bacterial bio-fermentation using modified organisms), molecular structure (linear, cross-linked or both), and method of cross-linking [106]. Furthermore, a paper has stated that, it is biologically reasonable to anticipate variations in the efficacy and adverse effect profiles between naturally occurring and cross-linked formulations [107,108]. Serious adverse events such as true septic arthritis are rare, with an incidence of approximately 0.08%, as reported in a large Danish study [108]. Risk factors for septic arthritis include advanced age, male sex, and pre-existing joint disease [108]. Prolonged exposure to corticosteroids raises concerns about chondral toxicity and accelerated disease progression. For example, a two-year trial of triamcinolone injections every three months demonstrated greater cartilage volume loss compared to saline injections where MRI revealed significantly greater cartilage volume loss (-0.21 mm vs -0.10 mm) in the steroid-treated cohort [109]. Additionally, repeated high-dose corticosteroid use has been linked to cytotoxic effects on chondrocytes and alterations in inflammatory pathways, necessitating cautious use [110]. Systemic effects include transient reductions in sex hormones and potential cardiovascular risks such as elevated blood pressure. Cases of osteonecrosis of the femoral head and other joints have also been reported, particularly in patients with predisposing factors like high cumulative steroid doses [111]. Transient hyperglycaemia and cushingoid appearances may occur with frequent injections, and up to 25% of patients may experience temporary HPA axis suppression, with cortisol levels returning to baseline within one-four weeks [111]. As an invasive procedure, IACS is subject to absolute and relative contraindications. Absolute contraindications include hypersensitivity to the injectate, significant skin breakdown, intra-articular fractures, systemic infections, septic arthritis, joint prostheses, severe joint destruction, and uncontrolled coagulopathy [112]. Relative contraindications are less clearly defined and require case-by-case clinical judgement [112]. 

The diminished effectiveness of repeated IACS injections (tachyphylaxis) in managing knee OA is a well-documented and widely observed phenomenon [98]. However, the underlying mechanisms behind this reduced response remain poorly understood. Several hypotheses have been proposed, drawing from analogous observations with similar medications or scattered reports in the existing literature. Further investigation is essential to establish whether these theories are directly implicated in the observed decline in efficacy with repeated injections. It is likely that this diminished response arises not from a single factor but from a combination of mechanisms. These may include receptor desensitisation, where the body's glucocorticoid receptors become less responsive to corticosteroids over time, as well as progressive joint degeneration that reduces the therapeutic impact of any intervention [79]. Other contributing factors may involve changes in pain processing mechanisms, such as central sensitisation, and alterations in the synovial membrane, such as fibrosis or diminished responsiveness to anti-inflammatory agents [95]. This multifactorial nature underscores the need for cautious and judicious use of steroid injections in knee OA. Additionally, it highlights the importance of exploring alternative or complementary treatment modalities to optimise patient outcomes and mitigate reliance on repeated corticosteroid injections.

To optimise patient outcomes, clinicians should tailor the selection of corticosteroid agents, dosing regimens, injection sites, and delivery methods to individual patient needs and clinical responses. Current recommendations suggest limiting corticosteroid injections to three or four per year for short-term symptom relief. However, larger, randomised controlled trials are needed to address unresolved questions regarding the efficacy, safety, and optimal use of IACS in knee OA management.

Biologics and cell-based/related therapies

Platelet-Rich Plasma

Platelet-rich plasma (PRP) is an increasingly common intra-articular therapy for knee OA. Platelets release numerous bioactive proteins and growth factors that influence inflammation, angiogenesis, cartilage metabolism, and tissue repair [9,113-115]. Over the past two decades, PRP has gained prominence for its safety and efficacy in mild-to-moderate knee OA, with meta-analyses showing superiority over placebo and other injectables, particularly at 6-12 months follow-up [116,117].

PRP formulations vary widely. Classification systems (e.g., leukocyte-rich vs. leukocyte-poor, PAW (platelet count, activation method, and white blood cell content) and DEPA (dose, efficiency, purity, and activation) criteria) reflect differences in leukocyte and fibrin content, preparation methods, and application [116,118,119]. This heterogeneity complicates comparisons, and although leukocyte-depleted PRP may offer superior outcomes, evidence remains inconsistent [116,120]. Emerging approaches include intraosseous delivery into subchondral bone, which may enhance results but requires further validation [121,122].

PRP is generally safe, with minor post-injection swelling or pain most common [116,123]. Future priorities include standardised protocols, optimised dosing, and combined use with adjuncts such as hyaluronic acid or mesenchymal stem cells (MSCs).

Mesenchymal Stem Cells

MSCs are multipotent precursors with regenerative and immunomodulatory effects [124-126]. Intra-articular administration promotes cartilage repair and symptomatic improvement through paracrine signalling, angiogenic and anti-inflammatory pathways, and creation of a supportive microenvironment [127-129]. Clinical evidence suggests benefits in pain, function, and imaging markers [130].

However, outcomes vary due to heterogeneity in cell source, processing, dosage, and protocols, with no consensus on the optimal approach [131]. Younger patients with early disease tend to respond better than those with advanced OA [132]. Minor local adverse events are reported, but long-term safety remains under study. Large randomised trials are needed to define best practice and evaluate synergy with other biologics such as PRP or hyaluronic acid.

Exosomes and Extracellular Vesicles

Exosomes are small, membrane-bound vesicles that mediate many MSC paracrine effects. They carry diverse biomolecules, including proteins, mRNAs, and particularly miRNAs, which regulate gene expression and promote cartilage repair [133-135]. Preclinical studies show they enhance cell proliferation, matrix deposition, and histological outcomes [133,134]. Their controlled delivery may address variability in MSC therapies and reduce immune risks, but clinical translation remains at an early stage. Further studies are required to clarify mechanisms, standardise production, and assess safety.

Autologous Micro-Fragmented Adipose Tissue

Autologous micro-fragmented adipose tissue (AMFAT), rich in stromal vascular fraction, has shown promising clinical results, improving pain and cartilage composition while slowing glycosaminoglycan loss [135,136]. Compared to bone marrow (BM)-derived MSCs, adipose tissue offers a higher cell yield per gram, though BM-MSCs may exhibit stronger chondrogenic potential in vitro. Combining adipose-derived MSCs with PRP appears synergistic in experimental studies. AMFAT is minimally invasive and safe, but direct comparative trials are still needed.

Disease-modifying osteoarthritis drugs

DMOADs are relatively new groups of medications which aim to inhibit cartilage degeneration, target key inflammatory/degenerative pathways [137]. These pathways include inhibition of cartilage and extracellular matrix degradation agents such as MMP-13 inhibitors and a disintegrin and metalloproteinase with thrombospondin motif (ADAMTS-5) inhibitors (MMP and ADAMTS are enzymes responsible for cartilage degradation/degeneration) [138]. While animal models highlight the therapeutic potential of these enzyme inhibitors targeting ECM degradation, clinical success remains elusive, necessitating further research to identify safer and more effective drugs for OA [137]. 

Other DMOADs target Wnt signalling pathways such loricivint and sprifermin [139]. The Wnt signalling pathway regulates chondrocyte differentiation and maintains metabolic balance in joints. In OA, activation of the Wnt/β-catenin pathway accelerates cartilage degradation and contributes to disease progression [139]. Key molecules like Wnt3a and β-catenin are upregulated, while antagonists like osteopenin are downregulated [139,140]. Targeting the Wnt pathway is a promising therapeutic approach. While some agents like loricivint and sprifermin have progressed to clinical trials, further studies are required to confirm efficacy and optimise their application, especially for early-stage disease and cartilage regeneration [137]. 

Cartilage repair promotion is another means by which some DMOADs act enhancing the limited self-healing capacity of the cartilage. For example, LNA043 is a derivative of angiopoietin-like 3 (ANGPTL3) that promotes cartilage formation and matrix synthesis [80]. Phase I and II trials demonstrate safety, with ongoing evaluation for efficacy [137,141]. Furthermore, Krüppel-like factor (KLF4) modulation enhances chondrogenic gene expression and suppresses inflammation. Mocetinostat, a selective histone deacetylase inhibitor, upregulates KLF4 and shows potential as a DMOAD [142]. The KLF is a family of transcription factors composed of 17 members in mammals and is involved in various biological and pathological mechanisms. Among them, KLF4 has been reported to enhance expression of chondrogenic genes in human chondrocytes, meniscal cells, and BM-derived MSCs (BMSCs), including SOX9, COL2A1 and ACAN, COMP, and PRG4 [142].

Inhibition of different inflammatory pathways and responses have also been the target of some disease-modifying osteoarthritis medication (DMOAM). Inflammation contributes to OA's onset and progression hence reducing inflammation can slow disease progression. Agents such as metformin have demonstrated anti-inflammatory and cartilage-protective effects by regulating adenosine monophosphate-activated protein kinase (AMPK) signalling and reducing leptin secretion [143]. Preclinical and observational studies suggest potential therapeutic benefits, particularly for obese OA patients [143]. Other medications, such as TPCA-1 and Tofacitinib (Tofa), target key inflammatory pathways, including IκB kinase and Janus kinase signalling, and have demonstrated the ability to protect cartilage from degradation in preclinical models of OA [144]. Future further studies are warranted to assess effectiveness [137]. 

Another group of the DMOAM agents is the ones that act on abnormal remodelling of subchondral bone in OA [145]. Subchondral bone changes, such as increased turnover, microfractures, and sclerosis, play a critical role in OA progression [145]. Addressing these abnormalities can improve joint function and delay OA progression [137]. There are few agents that are currently under different stages of assessment such as zoledronic acid which is a bisphosphonate that reduces bone resorption and cartilage degeneration in animal models [146]. Clinical trials show limited effects on cartilage volume loss, but further research continues [137]. Other agents such as MIV-711 which is a cathepsin K inhibitor that reduces biomarkers of bone and cartilage degradation, showing symptomatic improvement in phase II trials [137,146].

**Figure 4:**
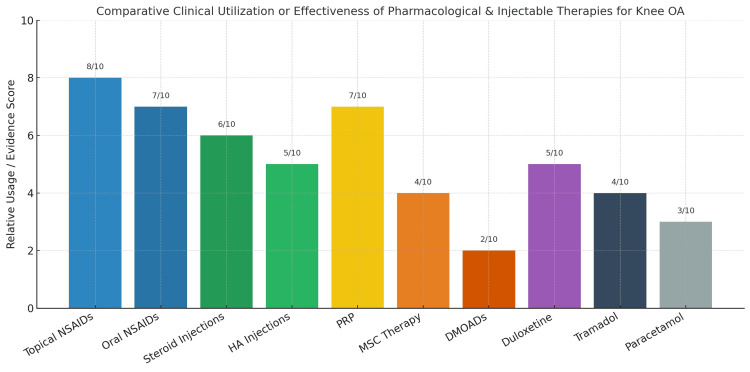
Comparative clinical utilisation or effectiveness of pharmacological and injectable therapies for knee OA Values are conceptual estimates based on published literature and current clinical guidelines [66-123]. OA: Osteoarthritis

Figure 4 provides a conceptual overview (on a 0-10 scale) of the relative utilisation and evidence supporting various pharmacological and injectable therapies commonly used for knee OA management. These relative scores were derived from contemporary clinical guidelines, meta-analyses, and expert consensus recommendations described in the present review [66-123].

Experimental and emerging new modalities for knee OA

Genicular Artery Embolisation

Angiogenesis and inflammatory mediators are key contributors to the pathogenesis of knee OA, as synovial inflammation induces neovascularisation and nerve growth, exacerbating pain and sensitisation [147]. Targeting these processes may offer a therapeutic approach to pain management [148]. Based on this premise, Okuno et al. introduced GAE using imipenem/cilastatin, demonstrating significant pain relief [149]. However, the limited global availability of this antibiotic restricts its clinical application [148]. Subsequent research has validated the efficacy of GAE with permanent microparticles, yet concerns persist regarding the risk of ischaemic complications with the permanent types of injections [148]. To mitigate these risks, the use of temporary embolic agents with well-established safety profiles has been proposed as a viable alternative [148].

Several case series have reported promising short-term outcomes for GAE in knee O; however, variations exist in indications, treatment protocols, and injection types among published studies. For example, recent investigations into the use of ethiodised oil-based emulsions in knee OA patients have demonstrated both safety and efficacy in alleviating symptoms [148]. Notably, patients with moderate to severe knee OA (KL grade 3-4) experienced substantial pain relief and functional improvement, with 73% meeting responder criteria and 41% achieving asymptomatic status within three months [148].

Despite these encouraging findings, GAE is not without risks. Reported complications include transient declines in renal function, likely attributed to the use of iodinated contrast agents for intra-arterial navigation, as well as potential occurrences of skin or bone ischemia [148]. Compared to alternative embolic materials, such as imipenem/cilastatin and permanent microparticles, ethiodised oil appears to offer a potential advantage by reducing the likelihood of non-target embolisation and its associated complications [148]. However, existing case series are limited by small sample sizes, the absence of randomised comparative groups, and short follow-up periods, necessitating a cautious interpretation of their findings. If further validated through larger, randomised controlled trials, GAE using an ethiodised oil-based emulsion could emerge as a viable, minimally invasive approach for managing knee OA-related pain and functional impairment.

Anti-obesity Medications

The utilisation of anti-obesity interventions, particularly pharmacological treatments, has garnered increasing attention in recent years. Newly approved semaglutide-based medications, such as Ozempic and Wegovy, have demonstrated potential benefits in addressing obesity-related conditions, including knee OA [150]. A well-established correlation exists between obesity and knee OA, with excess body weight significantly elevating the risk of developing the condition and exacerbating its associated symptoms [151]. Weight reduction is widely recognised as an effective strategy for both preventing OA and alleviating its clinical manifestations. Research indicates that reducing body weight can relieve pain and slow the progression of joint deterioration [151]. When combined with dietary modifications and physical activity, semaglutide has been identified as an effective pharmacological intervention for weight management, particularly among individuals experiencing metabolic challenges [150].

A paper has reported that semaglutide use is associated with a 16% reduction in OA risk among obese patients [151]. While these findings suggest a potential protective effect, they do not establish a causal relationship due to the lack of randomisation within the study design [150]. Furthermore, the study identified a 35% higher likelihood of OA development among female patients compared to males, while comorbid conditions such as gout, fibromyalgia, and migraine were also associated with an increased OA risk [150]. Notably, no significant differences in OA risk were observed between users of Ozempic and Wegovy, likely due to their shared active ingredient, semaglutide [150]. Although current evidence suggests an association between semaglutide use and a decreased likelihood of developing OA, further research is warranted to validate these findings. Future studies should incorporate randomised controlled trials to establish a definitive causal relationship between semaglutide use and OA risk reduction. Additionally, integrating more comprehensive diagnostic criteria, including the utilisation of biologics and OA-specific therapeutic interventions, may enhance the accuracy of patient identification and contribute to a deeper understanding of semaglutide's role in OA prevention and management.

Anabolic Agents/Fibroblast Growth Factors and Tissue Engineering

An increasing number of molecular factors and biological agents have demonstrated promising effects on various mechanisms involved in maintaining normal cartilage homeostasis and mitigating OA changes. These agents target critical cellular and molecular pathways, presenting potential therapeutic strategies to preserve cartilage integrity and slow the progression of OA. Cartilage homeostasis is a highly complex process that relies on a delicate balance between regenerative and degradative activities. Disruptions to this equilibrium can lead to cartilage degradation, a fundamental pathological feature of OA. By modulating specific molecular pathways, these emerging therapies hold significant potential to restore homeostasis and offer disease-modifying benefits in OA management.

Recent advancements in OA research have shifted focus toward exploring these biological agents capable of decelerating or reversing cartilage degradation [152-155]. Among these, fibroblast growth factor receptor (FGFR) antagonists have demonstrated significant promise in preclinical studies. The FGF family plays a crucial role in cartilage homeostasis, with different FGFs exerting distinct effects on cartilage metabolism [152]. While FGF2 and FGF8 are associated with catabolic responses, FGF9 and FGF18 promote anabolic processes [152,153]. Both FGFR1 and FGFR3 antagonists have shown encouraging outcomes in preventing cartilage degeneration and enhancing regeneration, highlighting their potential as future therapeutic strategies for OA [154]. Additionally, the expression of FGFR3 in chondrocytes decreases as OA progresses, suggesting that early administration of exogenous FGFs, particularly FGF9 and FGF18, could effectively stimulate FGFR3 signalling and provide more robust therapeutic effects in managing OA [155].

Beyond molecular therapies, integrating FGFs into tissue engineering approaches represents another promising strategy for OA treatment. Recent studies have utilised nanoscaffold technologies that mimic the extracellular matrix, combining them with growth factor treatments to support cartilage regeneration [156]. These nanoscaffolds, when combined with FGFs, have successfully facilitated the differentiation of human BM MSCs into chondrocytes, underscoring the potential of regenerative medicine in OA [156]. Overall, targeting FGF signalling pathways and employing advanced tissue engineering techniques could offer innovative and effective disease-modifying treatments for OA. Further research is necessary to elucidate the underlying molecular mechanisms and optimise these strategies for clinical application.

Bone Morphogenetic Proteins

Bone morphogenetic protein-7 (BMP-7), a member of the transforming growth factor-beta (TGF-β) superfamily, has exhibited significant anabolic and chondroprotective effects in vitro, providing a strong foundation for its potential therapeutic application in OA [157]. Preclinical studies have reinforced these findings, demonstrating beneficial effects of BMP-7 in animal models of OA [158]. Such studies suggest that BMP-7 may play a role in promoting cartilage regeneration and inhibiting the progression of OA, highlighting its potential as a disease-modifying agent. Beyond preclinical research, early clinical trials have assessed the safety profile of BMP-7 in the context of OA treatment. A Phase 1 double-blind, randomised, multicentre, placebo-controlled, single-dose escalation trial confirmed the safety of BMP-7, with no dose-limiting toxicity observed [159]. While these preliminary results are encouraging, further clinical trials are required to establish the efficacy of BMP-7 in managing OA. Additional research is necessary to validate these findings, optimise dosing strategies, and potentially incorporate BMP-7 into standardised therapeutic protocols for OA treatment.

Monoclonal Antibodies

Monoclonal antibodies (mAbs) represent an emerging therapeutic approach, with over 30 approved treatments for various diseases, including autoimmune disorders [160]. In the context of knee OA, mAbs have shown promising results in preclinical and animal models [160]. Several antibodies are currently being investigated in vitro, targeting various molecules within the knee joint, such as vascular endothelial growth factor (VEGF)-targeting antibodies (e.g., MF-1 and DC101) and tanezumab, an anti-nerve growth factor (NGF) antibody [160,161]. These mAbs have demonstrated significant improvements in pain and function in patients with moderate to severe knee OA. For instance, a Phase 3 study with the longest tanezumab treatment period (56 weeks) and safety follow-up period (24 weeks) revealed that tanezumab (2.5 mg and 5 mg) was more efficacious than NSAIDs in patients with moderate to severe knee or hip OA. However, rapidly progressive OA was more prevalent with the higher tanezumab dose, which prompted the US Food and Drug Administration (FDA) and European Medicines Agency (EMA) to express serious safety concerns regarding all anti-TNF clinical research [162,163].

Furthermore, immune response modulation has been explored in OA, with mAbs targeting cytokines such as TNF-α (e.g., adalimumab, infliximab, etanercept) and IL-1 (e.g., anakinra), showing some positive results, particularly in inflammatory OA phenotypes [161]. In conclusion, mAbs appear to present a favourable risk-benefit profile for OA treatment. However, given the multifactorial nature of OA, a more individualised therapeutic approach is necessary. Further research is required to confirm the efficacy of mAb therapy in specific patient phenotypes.

## Conclusions

Knee OA remains a major cause of pain and disability worldwide despite the range of available non-surgical treatments. While exercise, weight management, medications, and injections can reduce symptoms, they rarely halt disease progression.

Emerging therapies, such as PRP, stem cells, and GAE show promise but require further high-quality studies to clarify their long-term benefits. Ultimately, a personalised approach that matches treatment to each patient’s needs and disease stage will be essential for improving outcomes and delaying the need for joint replacement surgery.
